# Erdj3 Has an Essential Role for Z Variant Alpha‐1‐Antitrypsin Degradation

**DOI:** 10.1002/jcb.26069

**Published:** 2017-06-20

**Authors:** Nazli Khodayari, George Marek, Yuanqing Lu, Karina Krotova, Rejean Liqun Wang, Mark Brantly

**Affiliations:** ^1^ Division of Pulmonary, Critical Care and Sleep Medicine, Department of Medicine University of Florida Gainesville Florida

**Keywords:** ALPHA‐1‐ANTITRYPSIN, ERdj3, ER ASSOCIATED DEGRADATION, AUTOPHAGY

## Abstract

Alpha‐1‐antitrypsin deficiency (AATD) is an inherited disease characterized by emphysema and liver disease. AATD is most often caused by a single amino acid substitution at amino acid 342 in the mature protein, resulting in the Z mutation of the alpha‐1‐antitrypsin gene (ZAAT). This substitution is associated with misfolding and accumulation of ZAAT in the endoplasmic reticulum (ER) of hepatocytes and monocytes, causing a toxic gain of function. Retained ZAAT is eliminated by ER‐associated degradation and autophagy. We hypothesized that alpha‐1‐antitrypsin (AAT)‐interacting proteins play critical roles in quality control of human AAT. Using co‐immunoprecipitation, we identified ERdj3, an ER‐resident Hsp40 family member, as a part of the AAT trafficking network. Depleting ERdj3 increased the rate of ZAAT degradation in hepatocytes by redirecting ZAAT to the ER calreticulin‐EDEM1 pathway, followed by autophagosome formation. In the Huh7.5 cell line, ZAAT ER clearance resulted from enhancing ERdj3‐mediated ZAAT degradation by silencing ERdj3 while simultaneously enhancing autophagy. In this context, ERdj3 suppression may eliminate the toxic gain of function associated with polymerization of ZAAT, thus providing a potential new therapeutic approach to the treatment of AATD‐related liver disease. J. Cell. Biochem. 118: 3090–3101, 2017. © 2017 The Authors. *Journal of Cellular Biochemistry* Published by Wiley Periodicals Inc.

The endoplasmic reticulum (ER) is the site for synthesis and export of proteins. The ER contains molecular chaperones and folding enzymes that assist the newly synthesized proteins to fold and be transported from the ER [Braakman and Hebert, 2013]. ER chaperones and folding sensors, such as BiP, calnexin, and calreticulin, and co‐chaperones form a strict quality control (QC) system within the ER. The ER QC prevents the exit of folding intermediates and misfolded proteins from the ER by shuttling them to degradation pathways. Despite the complexity of the QC system and all the resources dedicated to protein folding, there are still many errors, which cause pathological conditions [Ellgaard and Helenius, 2003; Valastyan and Lindquist, 2014]. Mutations, insufficient chaperone availability, and aberrant folding are the most common causes for ER QC system failure and formation of misfolded molecules [Bross et al., 1999]. ER‐associated degradation (ERAD) is a collection of mechanisms that clear the ER of improperly folded proteins by targeting them for proteasomal degradation [Walter and Ron, 2011]. However, if these fail, unusual circumstances result in inappropriate aggregation of proteins and cellular stress, which can lead to a growing number of conformational diseases [Aguzzi and O'Connor, 2010].

Alpha‐1‐antitrypsin deficiency (AATD), a leading genetic cause of liver disease in children, is an inherited conformational disease, which is found in approximately 1:2000 live births [Fairbanks and Tavill, 2008]. Most adults with AATD demonstrate slowly progressive hepatic damage. Up to 50% of individuals with AATD will develop significant cirrhosis and occasionally some will develop hepatocellular carcinoma [Crowther et al., 2004]. A single amino acid substitution of lysine for glutamate at position 342 in the coding sequence of the SERPINA1 gene conduces the Z variant alpha‐1‐antitrypsin (ZAAT) molecule. ZAAT is the cause of PiZZ, the most common AATD variant, while MAAT is the most common normal AAT variant [Cox and Billingsley, 1989]. PiZZ (homozygous for ZAAT variant) results in polymerization of ZAAT in the ER of hepatocytes through insertion of the reactive center loop of one molecule into the β sheet of another protein molecule [Carrell and Lomas, 2002]. The responses of the PiZZ host to infection, inflammation, or trauma result in overexpression of AAT as an acute phase protein, which overwhelms the degradation pathway and leads to the formation of ZAAT inclusions in hepatocytes [Lomas, 2000]. In patients with AATD, accumulation of ZAAT molecules in hepatocytes reduces secretion of AAT by fivefold, predisposing deficient individuals to chronic obstructive pulmonary disease and emphysema [Kohnlein and Welte, 2008].

Reports have suggested that accumulation of ZAAT polymers within the ER of hepatocytes activates proteasomal and autophagic degradation pathways [Perlmutter, 2006; Kroeger et al., 2009; Gelling and Brodsky, 2010; Ghouse et al., 2014]. Chaperones interacting with ZAAT in the ER include calnexin, protein‐disulfide isomerase, GRP‐78/BiP, EDEM1, and the *N*‐glycan‐modifying enzymes UDP‐glucose: glycoprotein glucosyltransferase and α‐mannosidase‐like protein [Cabral et al., 2002; Schmidt and Perlmutter, 2005; Granell et al., 2008; Jang et al., 2015]. The balance between ZAAT polymer degradation versus folding and trafficking depends on stoichiometry of the ER chaperone system and degradation components [Adachi et al., 2008]. We hypothesized that proteostasis network components interacting with ZAAT in the ER play a critical role in targeting misfolded ZAAT for degradation. We demonstrate that ERdj3 co‐immunoprecipitates with ZAAT in Huh 7.5 cells. ERdj3 is a soluble, luminal glycoprotein that is a component of unassembled immunoglobulin (Ig) heavy chain. It is ubiquitously expressed, with the highest level of expression in secretory tissues, such as in the liver [Shen and Hendershot, 2005]. ERdj3 directly binds to nascent unfolded and misfolded proteins and transfers them to Hsp70 proteins, such as BiP [Guo and Snapp, 2013; Otero et al., 2014]. We demonstrate that depletion of ERdj3 reduces the accumulated ZAAT in the ER of the hepatocytes by enhancing ZAAT degradation. Furthermore, introducing siERdj3 to ZAAT‐expressing hepatocytes enhances degradation of ZAAT by autophagy and redirects ZAAT trafficking to the lectin ER chaperone calreticulin. We showed changes in ZAAT trafficking and its associated partners in normal and depleted expression of ERdj3 from the ER to the cytoplasm of hepatocytes. Finally, our data suggest that knocking down ERdj3 leads to formation of ZAAT‐containing, LC3‐positive autophagosomes, which might play a role in clearance of ZAAT from the ER of hepatocytes.

## MATERIALS AND METHODS

### CELL CULTURE AND TRANSFECTION

The AAT knockout (KO) Huh 7.5 cell line was created by using CRISPR/Cas9 and were cultured in DMEM/F12K supplemented with 10% fetal bovine serum and primocin TM (InvivoGen, San Diego, CA). Cells were transfected with NT (non targeting) siRNA or siERdj3 (Life Technologies, Carlsbad, CA) overnight using Lipofectamin RNAiMAX (Invitrogen, Waltham, MA) and transfected with pTR2‐ZAAT plasmid or pTR2‐MAAT using X‐tremeGENE HP DNA transfection reagent (Roche Applied Science, Indianapolis, IN) 24 h post silencing. Then, 48 h after transfection, cells were evaluated by Western blot, co‐immunoprecipitation (co‐IP), metabolic labeling, or immunofluorescence experiments. To investigate the effect of ERdj3 overexpression, AAT KO Huh7.5 cells were co‐transfected with GFP tagged pCMV6‐DNAJB11 (OriGene, Rockville, MD) and pTR2‐ZAAT or pTR2‐MAAT; 48 h post transfection, they were evaluated by native gel Western blotting to investigate the AAT polymer formation.

### ANTIBODIES AND REAGENTS

Rabbit polyclonal antibodies were used to detect ERdj3, EDEM1, and ERp57 (Proteintech, Chicago, IL); others were to detect AAT (DAKO, Carpentaria, CA), GAPDH (Santa Cruz, Dallas, TX), Lamp1, P62, LC3B (all from Cell Signaling, Danvers, MA), and calnexin and calreticulin (both from Stressgen biotechnologies, San Diego, CA). Bafilomycin A1, MG132, Brefeldin A, and mouse monoclonal anti‐β‐actin were purchased from Sigma (St. Louis, MO), and 2C1 against human AAT polymers was purchased from Hycult biotech (Netherlands). Mouse monoclonal antibodies against BiP and AAT were purchased from BD bioscience (San Jose, CA) and R&D systems (Minneapolis, MN), respectively. Alexa Fluor 488 goat anti‐mouse IgG and Alexa Fluor 594 goat anti‐rabbit IgG were purchased from Invitrogen. Primers, Dynabeads protein A and G, Superscript VILO cDNA synthesis kit, and ERdj3 siRNA were purchased from Life Technologies, and HP DNA transfection reagent and TaqMan Universal PCR Master Mix were purchased from Roche Applied Science. Disuccinimidyl suberate (DSS) cross‐linker was purchased from Thermo Fisher scientific (Waltham, MA).

### LIVE/DEAD VIABILITY ASSAY

Cell viability was determined in the presence of NTsiRNA or siERdj4 using viability/cytotoxicity kit from Invitrogen following the manufacturer's protocol. Briefly, AAT KO Huh7.5 cells transfected with NTsiRNA or siERdj4 were cultured on 96 well assay plate for 1 day. Twenty‐four hour post silencing they transfected with ZAAT plasmid and grown until acceptable cell density. Cells with no treatment considered as live control and 0.1% saponin was added to the cells for 10 min for dead cells control. Four micrometer of Ethidium homodimer‐1 (EthD‐1) and 2 µM of Calcein AM were added to each well. The plate was then incubated at room temperature for 45 min. Absorbance at 530 and 645 nm was measured and recorder using ELISA microplate reader.

### METABOLIC LABELING STUDIES

Nearly confluent monolayers of AAT KO Huh 7.5 cells were transfected with NT siRNA or siERdj3. Then, 24 h post silencing, eukaryotic expression vectors expressing ZAAT or MAAT were introduced into 35‐mm diameter culture dishes. Forty‐eight hour post transfection they were incubated with or without 10 µM MG132 or 100 µM bafilomycin A1 for 6 h and the same amount of DMSO as control. The cells were incubated for 10 min with [^35^S] methionine (200–500 μCi/mL of medium; PerkinElmers, Waltham, MA) [Novoradovskaya et al., 1998] and then chased for 0–4 h by incubation in 1 mL of DMEM/F12 with 10% fetal bovine serum containing a fivefold excess of unlabeled methionine. Cells were harvested in a total volume of 1 mL IP lysis buffer (Pierce, Waltham, MA), kept on dry ice, and centrifuged at 15,000*g* for 5 min at 4°C to precipitate cell debris. ZAAT or MAAT was immunoprecipitated from the cell lysate and medium using rabbit anti‐human AAT antibody bound to protein A Dynabeads. Imunocomplexes were washed, suspended in 20 μL of sample buffer, heated at 70°C for 10 min, and analyzed using SDS Tris‐Glysin 10% PAGE (Bio‐Rad, Hercules, CA). Radiolabeled AAT was detected by autoradiography.

### ERdj3 ELISA ASSAY

Plasma samples were obtained from the Alpha‐1 Foundation DNA and Tissue Bank at the University of Florida (IRB #201500842) and stored in −80°C. Samples were from 11 normal individuals (MM) and 24 individuals with AATD whose livers contained periodic acid–schiff‐positive, diastase‐resistant globules within hepatocytes, with score from 0 to 3 on a 3‐point scale, in which 0 represents no liver disease and three represents severe disease. Samples were subjected to commercially available ELISA kit (MyBioSource, San Diego, CA) to determine the levels of ERdj3, according to manufacturer's instruction. ERdj3 levels were expressed as nanogram per milliliter of plasma.

### POLYMER FORMATION ASSAY

AAT KO Huh7.5 cells were co‐transfected with ERdj3 and ZAAT or MAAT plasmids. Forty‐eight hour post transfection, the cells were washed three times with PBS and lysed in PBS containing 100× Halt protease inhibitor cocktail (Thermo scientific). Lysates were vortexed for 2 min, followed by centrifugation at 18000*g* for 5 min at 4°C. Half of the supernatants were incubated at 37°C and half at 60°C for 1 h to form polymers. Total protein was resolved on non‐denaturing Mini Protein Gel TGX 10% (Bio‐Rad) using Tris glycine pH 8.9 and 0.1 M Tris pH 7.8 buffering systems. Proteins were transferred to nitrocellulose membranes. The blots were blocked and incubated with rabbit polyclonal AAT antibody. AAT polymers were detected with a Super Signal West Dura Extended Duration Substrate Kit (Thermo Scientific).

### DENSITY GRADIENT ISOLATION OF CELLULAR PROTEINS

AAT KO Huh 7.5 cells were transfected with NT siRNA or 20 nM of siERdj3 to silence ERdj3. Twenty‐four hour post silencing the cells were transiently transfected with ZAAT. Forty‐eight hour after transfection with ZAAT, the cells were incubated with or without 100 µM bafilomycin A1 for 6 h and were washed with 1× PBS to remove the media and debris. Next, 2 mL of cold isotonic buffer (250 mM sucrose, 10 mM TEA_AC, 1 mM EDTA) was added to 10‐cm dishes on ice, and cells were scraped into the buffer and transferred to a 15‐mL tube and then centrifuged at 15000*g* for 5 min. The pellet was resuspended and homogenized in 300 µL of hypotonic buffer (80 mM sucrose, 10 mM TEA_AC, 1 mM EDTA) and 100× Halt protease inhibitor cocktail and diluted in 300 µL of hypertonic buffer (420 mM sucrose, 10 mM TEA_AC, 1 mM EDTA). The cell lysate was centrifuged at 3000*g* for 5 min, and the supernatant was collected and inserted into the step gradient composed of 2.5–30% iodixanol solutions in 14‐mL ultra‐clear tubes (Beckman Coulter, Brea, CA). Then, within 1 h, the tubes were ultra‐centrifuged at 90,000*g* for 1 h at 4°C (SW40Ti rotor, Beckman Coulter). After centrifugation, 11 fractions were collected and stored at −20°C until analysis.

### IMMUNOBLOTTING (IB) AND IMMUNOPRECIPITATION (IP)

To investigate the level of ZAAT expression, AAT KO Huh 7.5 cells were seeded at 3 × 10^5^ /well in 6‐well plates with RNAiMAX‐siERdj3 complex. Then, 24 h post silencing, ZAAT plasmid was transfected using X‐TremeGENE HP DNA transfection reagent. Transfected cells were collected 48 h post transfection and lysed in RIPA buffer containing 50 mM Tris, 150 mM NaCl, 0.5% SDS, 0.5% sodium deoxycholate, and 1% NP‐40. Protein levels in the cell lysate homogenates were determined using a commercially available kit according to the bicinchoninic acid (BCA) method (Pierce Biotechnology, Rockford, IL). Total protein was resolved on Tris glycine SDS–PAGE gels (Bio‐Rad). Proteins were transferred to nitrocellulose membranes. The blots were blocked and incubated with indicated antibodies overnight at 4°C. Proteins were detected by using a Super Signal West Dura Extended Duration Substrate Kit (Thermo Scientific). Western blot band intensities were quantified using Alpha view software (ProteinSimple, San Jose, CA).

To examine the interaction of ZAAT with ER‐localized chaperones, co‐IP reactions were performed with antibodies against AAT, calreticulin, CNX, ERp57, BiP, EDEM1, and ERdj3. Before IP, half of each sample was cross‐linked by resuspending it in 25 mM DSS in PBS and incubating the preparation at room temperate for 30 min. Cross‐linked and non‐cross‐linked cells were washed with cold PBS and lysed in 0.5 mL of IP buffer. Approximately 1 µg of AAT antibody was added to 45 µL of protein A Dynabeads. After incubation for 10 min at room temperature, the protein A was washed for 3 min with wash buffer (0.1% Triton X‐100 in PBS). The lysates were incubated with anti‐AAT‐conjugated beads overnight at 4°C. The next day, beads were washed several times in wash buffer, then resuspended in gel loading buffer, and evaluated by Western blot using antibodies against different chaperones. To investigate if ERdj3 interacts with MAAT, co‐IP was performed with MAAT transiently transfected in AAT KO Huh 7.5 cells. 0.1, 0.25, and 0.5 µg of MAAT plasmid were introduced to AAT KO Huh 7.5 followed by 3h incubation with 20 µM of Brefeldin A and the ERdj3 associated with MAAT was determined with co‐IP using Dynabeads conjugated with rabbit antibody against AAT in each three samples.

### IMMUNOSTAINING, IMMUNOFLUORESCENCE MICROSCOPY, AND IMAGE ANALYSIS

Untreated, NT siRNA or siERdj3 treated AAT KO Huh7.5 cells were grown on glass coverslips and transfected with ZAAT or MAAT‐RFP plasmid. A 48 h post transfection, the cells were fixed with 4% paraformaldehyde in PBS for 20 min. The coverslips were washed with 1× PBS. The cells were incubated for 1 h with blocking buffer (1× PBS, 5% goat serum, and 0.3% Triton X‐100) at room temperature, followed by incubating overnight in 4°C with primary antibodies (1:400). The cells were washed with 1× PBS and incubated for 1 h with secondary antibodies (Alexa Fluor 488 goat anti‐mouse IgG and Alexa Fluor 594 goat anti‐rabbit IgG). The coverslips were mounted and sealed. Images were collected using a fluorescence microscope (Nikon, Melville, NY). Samples were scanned with a 0.1‐µm step. Images were processed for brightness and contrast and filtered for noise with Volocity 6.3 software (Perkin Elmer, Waltham, MA) following good practices as outlined by Rossner and Yamada [2004].

### STATISTICAL ANALYSIS

All results are expressed as mean ± S.E. Statistical analysis was performed using Prism 6 software program (GraphPad Software). Values of *P* < 0.05 was considered statistically significant.

## RESULTS

### ERdj3 INTERACTS WITH AAT IN HEPATOCYTES AND INCREASES ZAAT POLYMER FORMATION

**Figure 1:**
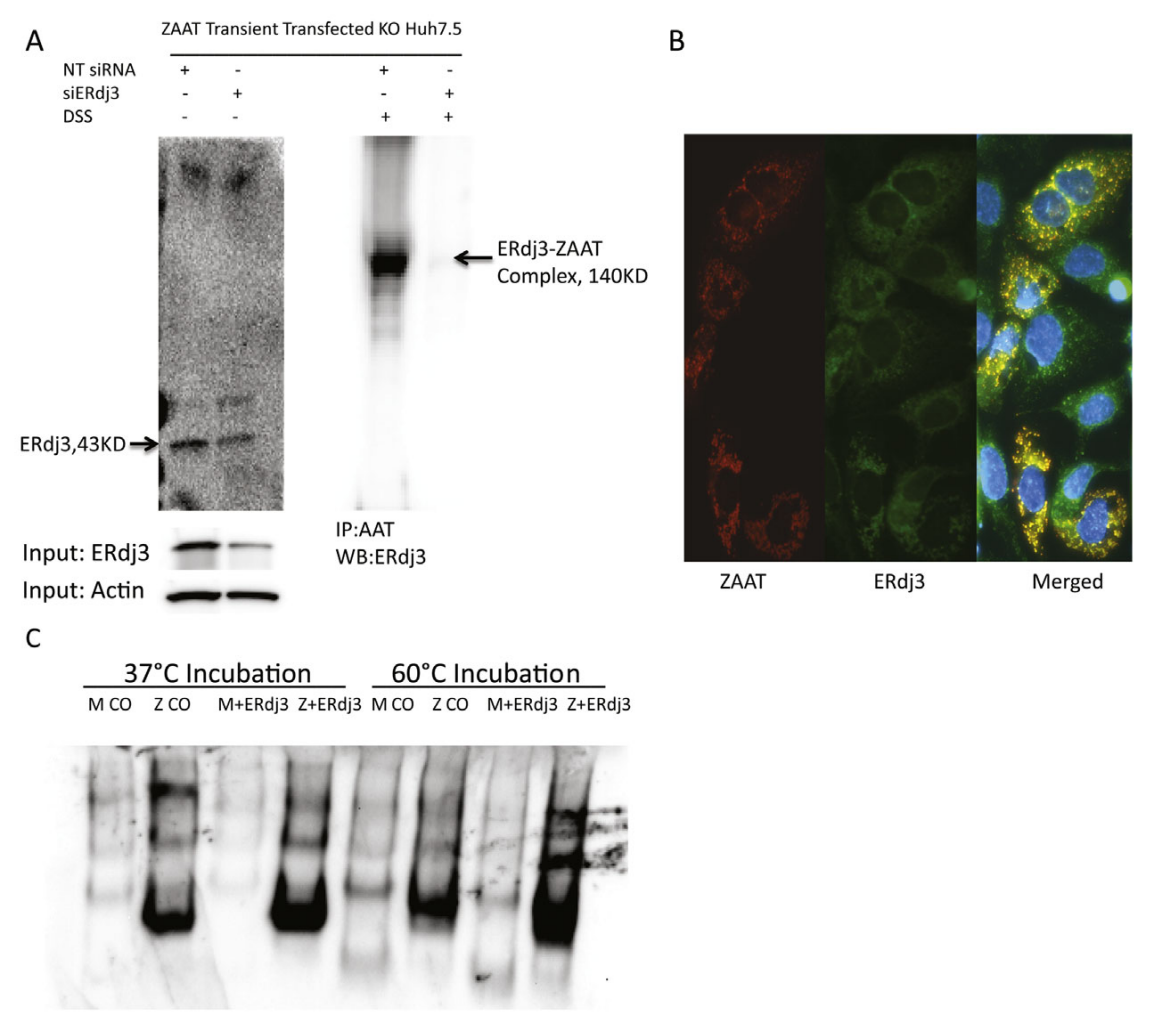
Interaction of ERdj3 with ZAAT. (A) ZAAT co‐immunoprecipitated with ERdj3 with and without cross‐linker. AAT KO Huh 7.5 cells were transfected with NT siRNA or siERdj3. Then, 24 h post silencing, cells were transfected with ZAAT plasmid. AAT was pulled down, and ERdj3 bound to ZAAT was determined by Western blot. (B) ERdj3 co‐localizes with ZAAT polymers. ZAAT transiently transfected cells were immunostained using 2C1 antibody (Alexa 594; red) and anti‐ERdj3 (Alexa 488; green), and nuclei were stained using DAPI (blue). (C) Erdj3 overexpression increases ZAAT polymer formation. MAAT and ZAAT transiently transfected cells were transfected with control (CO) or ERdj3 plasmids 24 h after the first transfection. Then, 48 h post transfection, lysates were incubated for 1 h at 37°C or 60°C to form polymers. Lysates were loaded on non‐denaturing native gel.

Several chaperones interact with ZAAT in the ER [Cabral et al., 2002; Schmidt and Perlmutter, 2005]. ERdj3 is one of the seven ER J domains–containing Hsp40 co‐chaperones that interact with unfolded and misfolded proteins [Yu et al., 2000]. To examine ZAAT interaction with ERdj3 within the ER of hepatocytes, we performed a co‐IP experiment. In support of the hypothesized role for ERdj3 in ZAAT trafficking, this chaperone was found to interact with ZAAT both in the presence and absence of a cross‐linker in AAT KO Huh 7.5 cells expressing ZAAT (Fig. 1A). To investigate if ERdj3 also interacts with MAAT, co‐IP was performed with MAAT transiently transfected in AAT KO Huh7.5 cells. In all three amounts of MAAT plasmid used, we observed an association between MAAT and ERdj3 (Fig. S2A). Likewise, ZAAT polymers co‐localized with ERdj3, as well as MAAT‐RFP, in the ER of hepatocytes in immunofluorescence experiments (Figs. 1B and S2B). To investigate the role of ERdj3 in ZAAT trafficking, we performed native gel Western blotting using AAT KO Huh 7.5 cells co‐transfected with ERdj3 and ZAAT or MAAT plasmids; 48 h post transfection, we observed AAT polymer formation. MAAT did not form polymers in the presence or absence of exogenous ERdj3 under any of the temperature conditions. Surprisingly, gene transfer‐mediated overexpression of ERdj3 enhanced ZAAT polymer formation at both 37°C and 60°C (Fig. 1C). Induced polymer formation can be attributed to the delay in ZAAT degradation. In a previous report, prolonged association of ERdj3 with substrates might delay their degradation [Shen and Hendershot, 2005].

### DEPLETION OF ERdj3 ENHANCES AAT DEGRADATION IN HUH 7.5 CELL LINE

**Figure 2:**
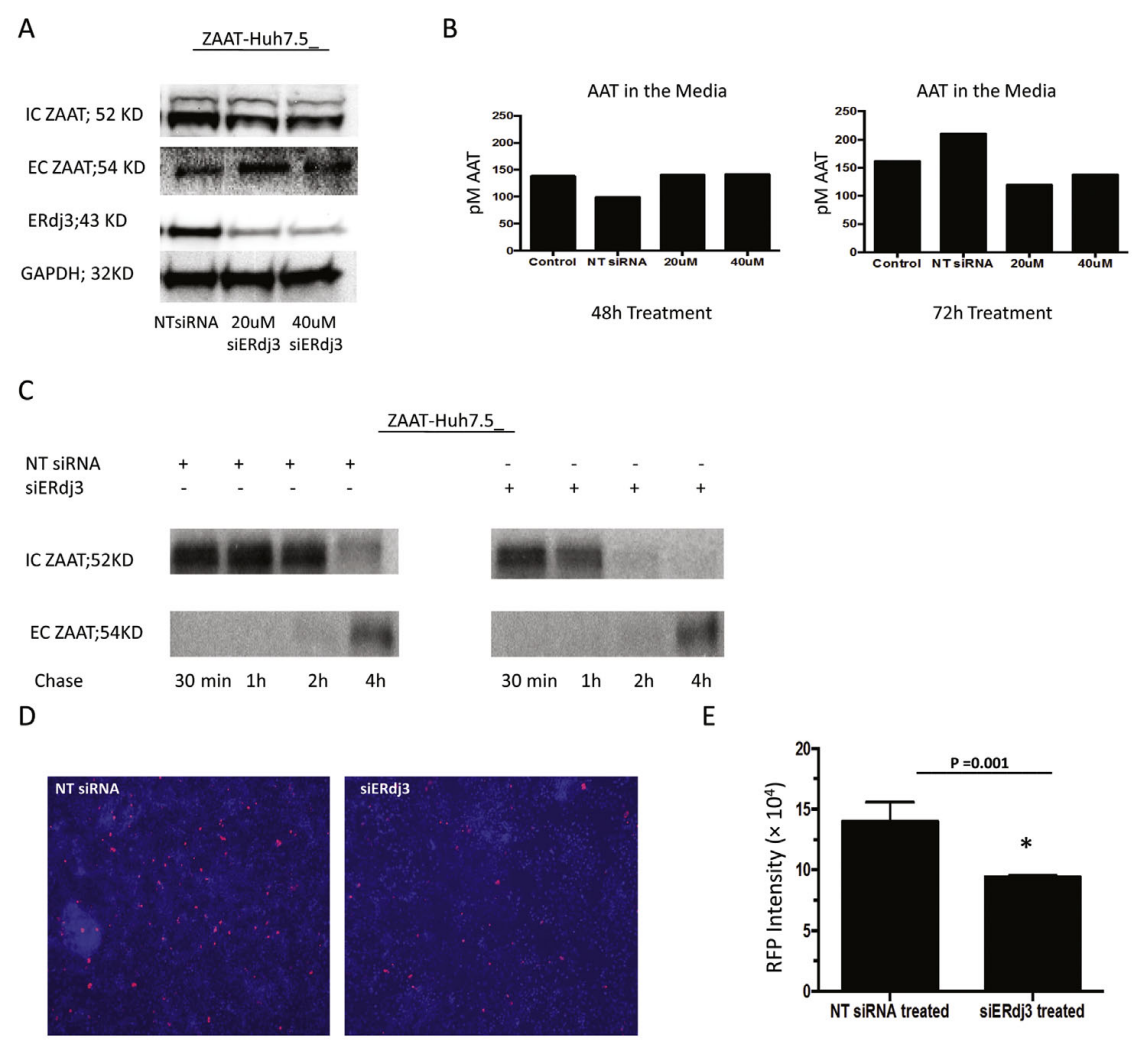
The effect of siERdj3 on ZAAT degradation. (A) siERdj3 increases ZAAT degradation. NT siRNA or siERdj3 (20 or 40 µM) were introduced 24 h post ZAAT transfection to AAT KO Huh 7.5 cells. The level of ZAAT inside the cells (IC) and in the media (EC) and the level of ERdj3 were measured by Western blot. GAPDH was used as loading control. (B) siERdj3 does not affect ZAAT secretion. By ELISA, total AAT was measured in the media from the same samples as in panel A experiment. Total AAT was shown in picomolar concentration. (C) siERdj3 accelerates ZAAT clearance from the ER of hepatocytes. NT siRNA or 20 nM of siERdj3 was introduced 24 h post ZAAT transfection to AAT KO Huh 7.5 cells. IC ZAAT and EC ZAAT from NT siRNA and siERdj3‐treated samples were shown after pulse‐chase radiolabeling. (D) ZAAT polymers are reduced following siERdj3 treatment. NT siRNA or 20 µM of siERdj3 were introduced 24 h post ZAAT transfection to AAT KO Huh 7.5 cells. ZAAT polymers were immunostained with 2C1 antibody (Alexa 594; red). (E) Integrated density of red fluorescent measurement from four images per sample.

Hsp40s, such as ERdj3, facilitate degradation and act as a chaperone independently of their analogous Hsp70s [Buck et al., 2010; Kampinga and Craig, 2010]. It can also over stabilize the interactions of chaperone and substrate, delaying the degradation of substrate [Shen and Hendershot, 2005]. To determine the role of ERdj3 in the AAT trafficking pathway, we used siRNA to silence ERdj3. In a preliminary live‐dead assay, we observed cell viability in ERdj3 knockdown cells, and silencing ERdj3 did not reduce cell viability (Fig. S1). Next, AAT KO Huh 7.5 cells were transfected with NT siRNA or siERdj3 followed by ZAAT transfection. A 48 h after transfection with ZAAT, total lysate and media were subjected to western blot analysis using anti AAT and anti ERdj3 rabbit polyclonal antibodies. The volume of media to detect extracellular (EC) ZAAT was correlated to the total protein in the corresponding cell lysate. Transfection of siERdj3 into hepatocytes efficiently suppressed approximately 70% of the endogenous ERdj3 protein level, which resulted in intracellular (IC) ZAAT degradation with no changes in EC ZAAT level (Fig. 2A). The media from the same experiment were subjected to AAT ELISA in 48 and 72 h post silencing to determine changes in the ZAAT secretion level. Compared with the control, no significant changes were observed in the level of secreted ZAAT in ERdj3‐depleted cells (Fig. 2B). To evaluate the level of ZAAT degradation, a similar experiment was performed using ^35^S pulse‐chase analysis. Silencing ERdj3 considerably enhances the level of IC ZAAT degradation, as well as MAAT degradation, in a 4‐hour chase period in ZAAT‐ and MAAT‐expressing Huh7.5 cells (Figs. 2C and S2C). The results were confirmed with immunoflourscent microscopy using 2C1 anti‐mouse antibody against ZAAT polymers in NT siRNA‐ and siERdj3‐transfected cells (Fig. 2D). Comparing integrated density of red fluorescent measurement from four 200× images per sample revealed a considerable reduction in ZAAT polymer staining in depleted ERdj3 cells compare with the control (Fig. 2E). To determine if siERdj3 affects AAT at the RNA level, qPCR was performed for AAT and major unfolded protein response (UPR) related genes. No significant changes were observed in the presence of siERdj3 compare with NT siRNA‐treated cells in terms of AAT RNA level and UPR genes expression. By Western blot analysis, compared with the NT siRNA control, the protein levels of major ER chaperones did not change in the presence of siERdj3 (Fig. S3).

### MG132 AND BAFILOMYCIN INHIBIT ERdj3‐MEDIATED ZAAT DEGRADATION

**Figure 3:**
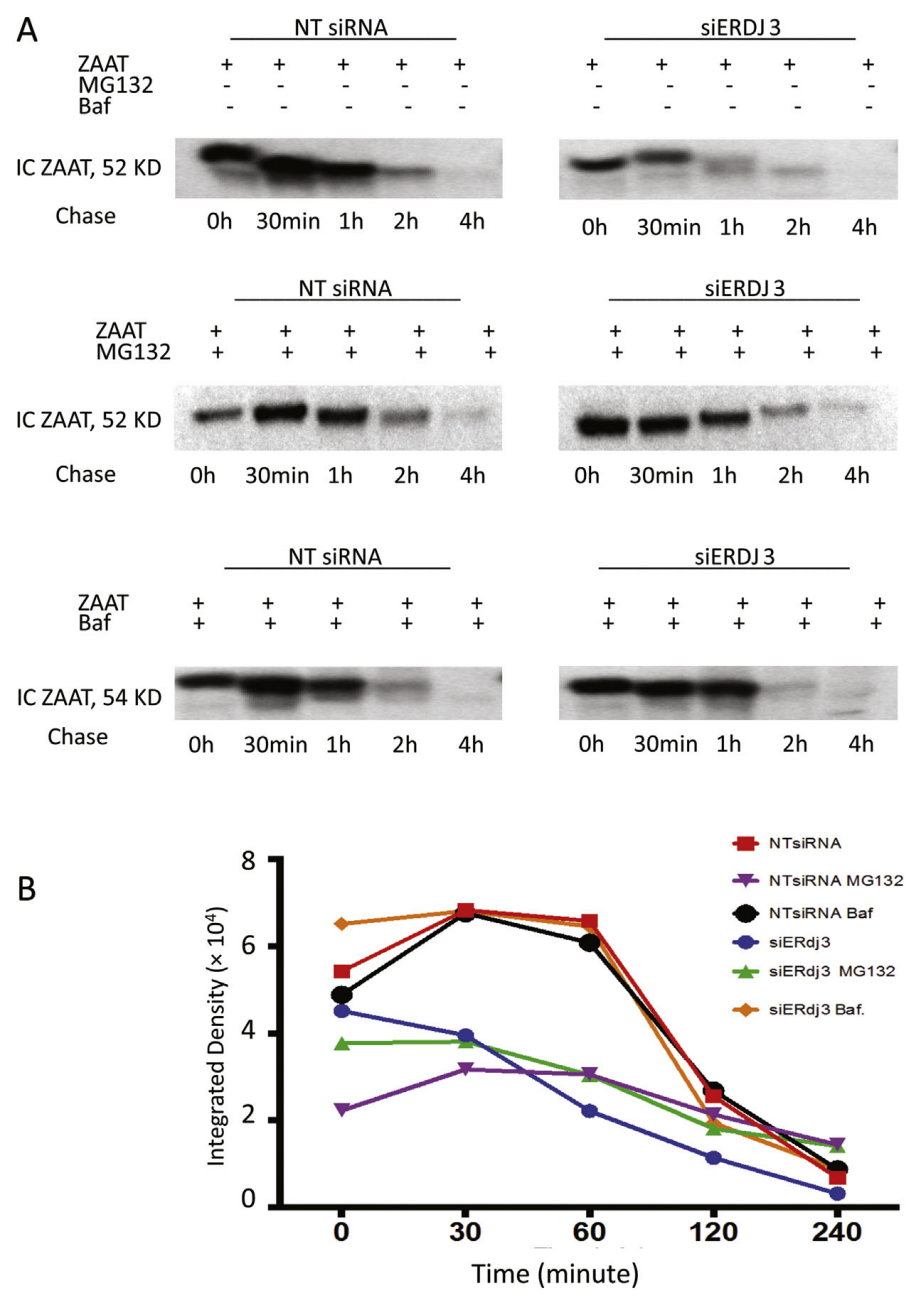
The effect of MG132 and bafilomycin on ERdj3‐mediated ZAAT degradation. (A) MG132 and bafilomycin inhibit ERdj3‐mediated ZAAT degradation. IC ZAAT from NT siRNA‐ and siERdj3‐treated ZAAT‐expressing samples were shown after pulse‐chase radiolabeling during 4‐h chase period. Cells were treated with MG132 or bafilomycin A1 6 h before labeling. (B) IC ZAAT quantified integrated density bars during 4‐h chase period.

We used bafilomycin A1 as an inhibitor of lysosomal degradation and MG132 as an established proteasome inhibitor [Yuan et al., 2015]. To clarify which pathway is being activated in siERdj3‐mediated ZAAT degradation, ^35^S pulse‐chase experiments were performed using NT siRNA and siERdj3 treatment on ZAAT expressing KO Huh 7.5 cells. Consistent with previous experiments, siERdj3 enhanced ZAAT degradation during the 4‐h chase period. Bafilomycin greatly inhibited ERdj3‐mediated ZAAT degradation; compared with this, MG132 affected ERdj3‐mediated ZAAT degradation less (Fig. 3A). Quantified integrated density bars illustrate the inhibitory effect of MG132 and bafilomycin on siERdj3‐mediated ZAAT degradation during the 4‐h chase period (Fig. 3B).

### ERdj3 DEPLETION ENHANCES AUTOPHAGIC RESPONSE TO ZAAT ACCUMULATION

**Figure 4:**
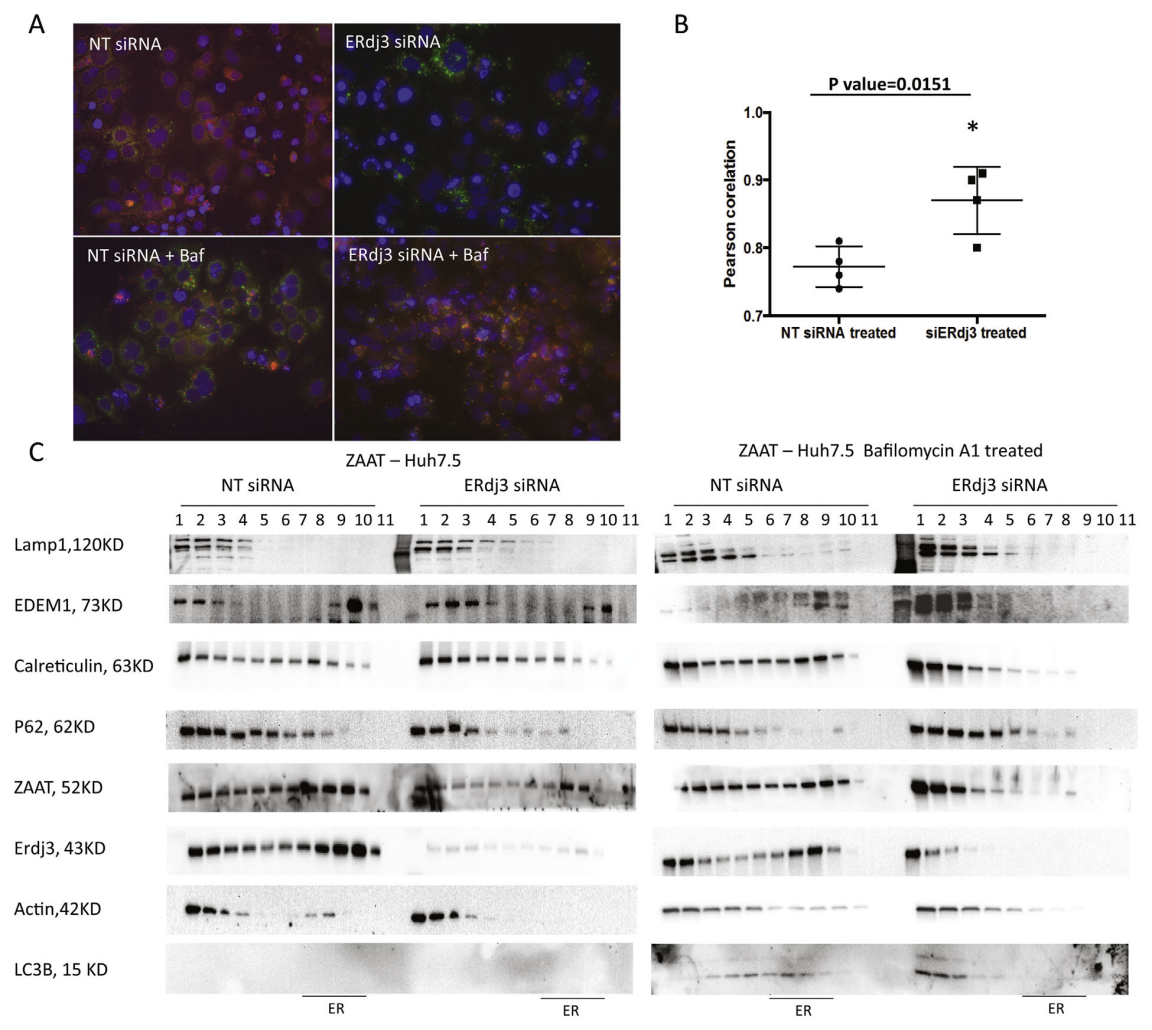
Increased autophagic response to ZAAT accumulation mediated by ERdj3 depletion. (A) LC3‐positive autophagosomes co‐localize with ZAAT in presence of bafilomycin, upon siERdj3 treatment. NT siRNA or 20 µM of siERdj3 was introduced 24 h post ZAAT transfection to AAT KO Huh 7.5 cells. Then, 48 h later, the cells were treated with or without bafilomycin for 6 h. ZAAT was immunostained using mouse anti‐AAT antibody (Alexa 488; red) and rabbit anti‐LC3 (Alexa 594; green). (B) Pearson correlation coefficients graph from four different images with siERdj3‐ versus NT siRNA‐treated samples. (C) Density gradient isolation of cellular proteins from the ZAAT‐expressing cells treated with NT siRNA or siERdj3 in presence and absence of Bafilomycin A1 shows that siERdj3 activates autophagic response to ZAAT accumulation. siERdj3 and bafilomycin A1 treatment results in ZAAT appearance in light‐density fraction, accompanied by EDEM1 and calreticulin in LC3‐ and Lamp1‐positive compartments.

Autophagy is a major IC degradation pathway mediated by Atg family proteins. The association of autophagic responses with degradation of ZAAT has been previously clarified [Hidvegi et al., 2005; Kamimoto et al., 2006]. We hypothesized that ERdj3 depletion allows ZAAT to be removed by autophagosomes. To test this, we characterized ZAAT subcellular distribution by fluorescent microscopy. In an immunofluorescence analysis of ZAAT‐expressing Huh 7.5 cells treated with NT siRNA or siERdj3 in the presence and absence of bafilomycin A1, ERdj3 depletion exhibited a puncta pattern in the cells regardless of bafilomycin treatment. Cells treated with siERdj3 displayed greatly less red fluorescent signal, which represents less ZAAT accumulation (Fig. 4A). Bafilomycin A1 treatment for 6 h led to co‐localization of ZAAT with endogenous LC3B in the cells with depleted ERdj3, suggesting that ZAAT is in vesicles (Fig. 4A). Silencing ERdj3 resulted in increased co‐localization of ZAAT with endogenous LC3B. The mean Pearson correlation coefficients were significantly different (*P* = 0.0151) for siERdj3‐ versus NT siRNA‐treated samples (Fig. 4B). To confirm this observation, we performed density gradient isolation of cellular proteins from the ZAAT‐expressing cells treated with NT siRNA or siERdj3 in the presence and absence of bafilomycin A1. Treatment with siERdj3 alone resulted in reduction of accumulated ZAAT (Fig. 4C). Furthermore, calreticulin and EDEM1 migrated from the ER to the light‐density fraction upon reduction of ERdj3. These light‐density fractions express Lamp1, which is an established marker for lysosomal and autophagolysosomal vesicles [Eskelinen et al., 2002]. However, LC3B was not detected in the absence of bafilomycin A1. In the presence of bafilomycin A1 and siERdj3, ZAAT appeared in the lighter fraction, with the same pattern as Lamp1, calreticulin, EDEM1, and endogenous LC3B. Altogether, our data therefore demonstrate that in the presence of siERdj3, ZAAT localized in autophagic structures; furthermore, such localization with ER‐resident chaperones may be important for ZAAT degradation.

### REDUCED INTERACTION BETWEEN ZAAT AND ERdj3 DIRECTS ZAAT TRAFFICKING TO THE CALRETICULIN PRO‐FOLDING PATHWAY

**Figure 5:**
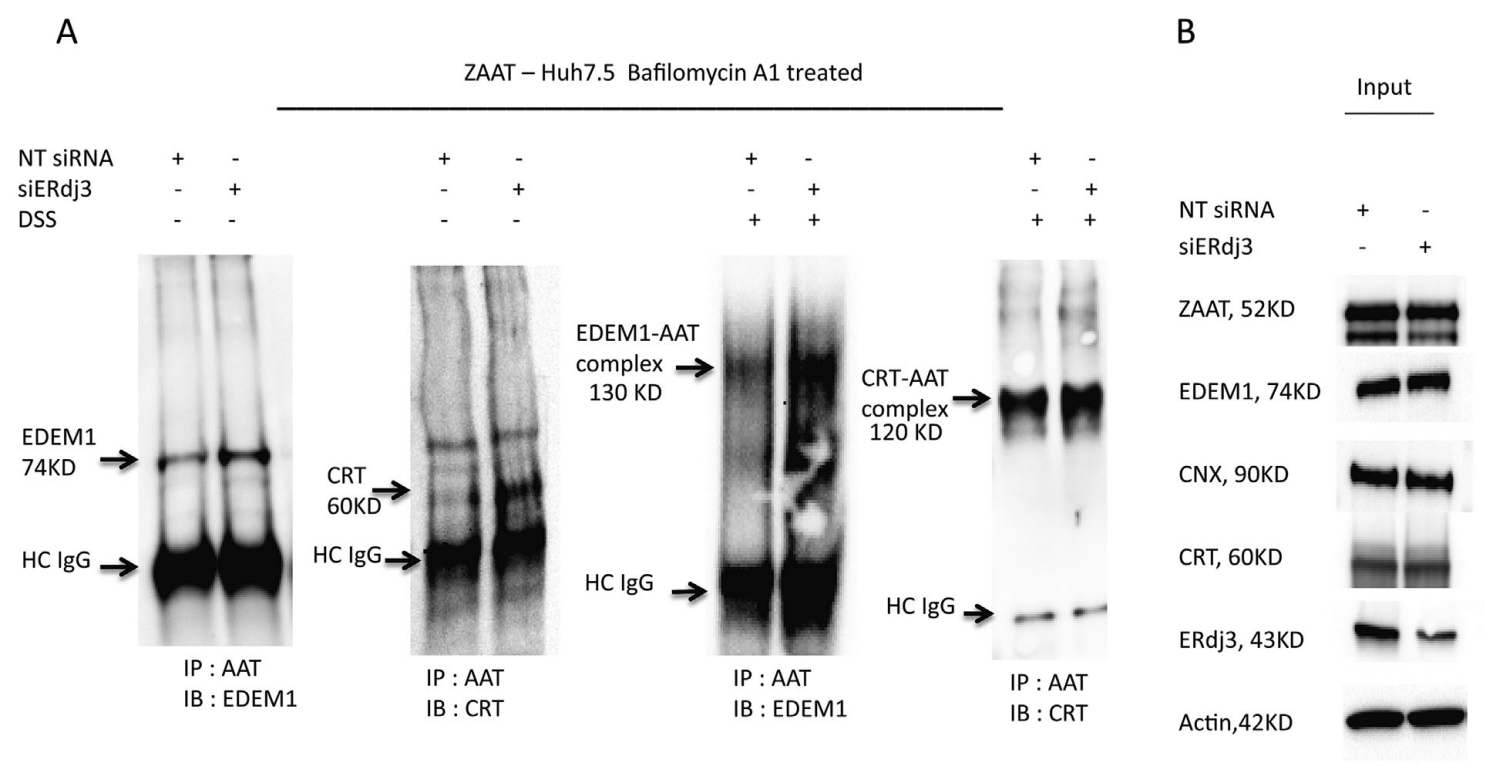
Activation of calreticulin pro‐folding pathway mediated by reduced interaction between ZAAT and ERdj3. (A) ERdj3 depletion results in ZAAT enhanced association with calreticulin and EDEM1. NT siRNA or 20 µM of siERdj3 was introduced 24 h post ZAAT transfection to AAT KO Huh 7.5 cells, followed by 6 h of Bafilomycin A1 treatment. The ZAAT association with calreticulin and EDEM1 was detected with and without DSS using co‐IP. (B) The total levels of ZAAT, EDEM1, calnexin, calreticulin, and ERdj3 were detected as input using Western blot analysis.

Because silencing of the ERdj3 co‐chaperone in the ER resulted in ZAAT degradation, we hypothesized that ERdj3 competes with the calnexin/calreticulin pro‐folding pathway. We performed IP analysis to identify ZAAT‐interacting proteins in the presence and absence of ERdj3. When the concentration of ERdj3 was reduced, more calreticulin and EDEM1 were bound to immunoprecipitated ZAAT, indicating that ZAAT was being conducted to the calreticulin pathway (Fig. 5). Consistent with previous data [Rutkevich et al., 2010], we could not detect any interaction between ZAAT and Erp57 (data not shown). ERp57 interacts with calnexin and calreticulin and contributes to their function as molecular chaperones by catalyzing the formation of disulfide bonds in substrates [Ellgaard and Frickel, 2003]. Despite some reports [Wu et al., 1994; Qu et al., 1996; Liu et al., 1997], we were unable to detect ZAAT in association with calnexin or BiP.

### ERdj3 ASSOCIATES DIRECTLY WITH CALRETICULIN IN HUH 7.5 CELLS

**Figure 6:**
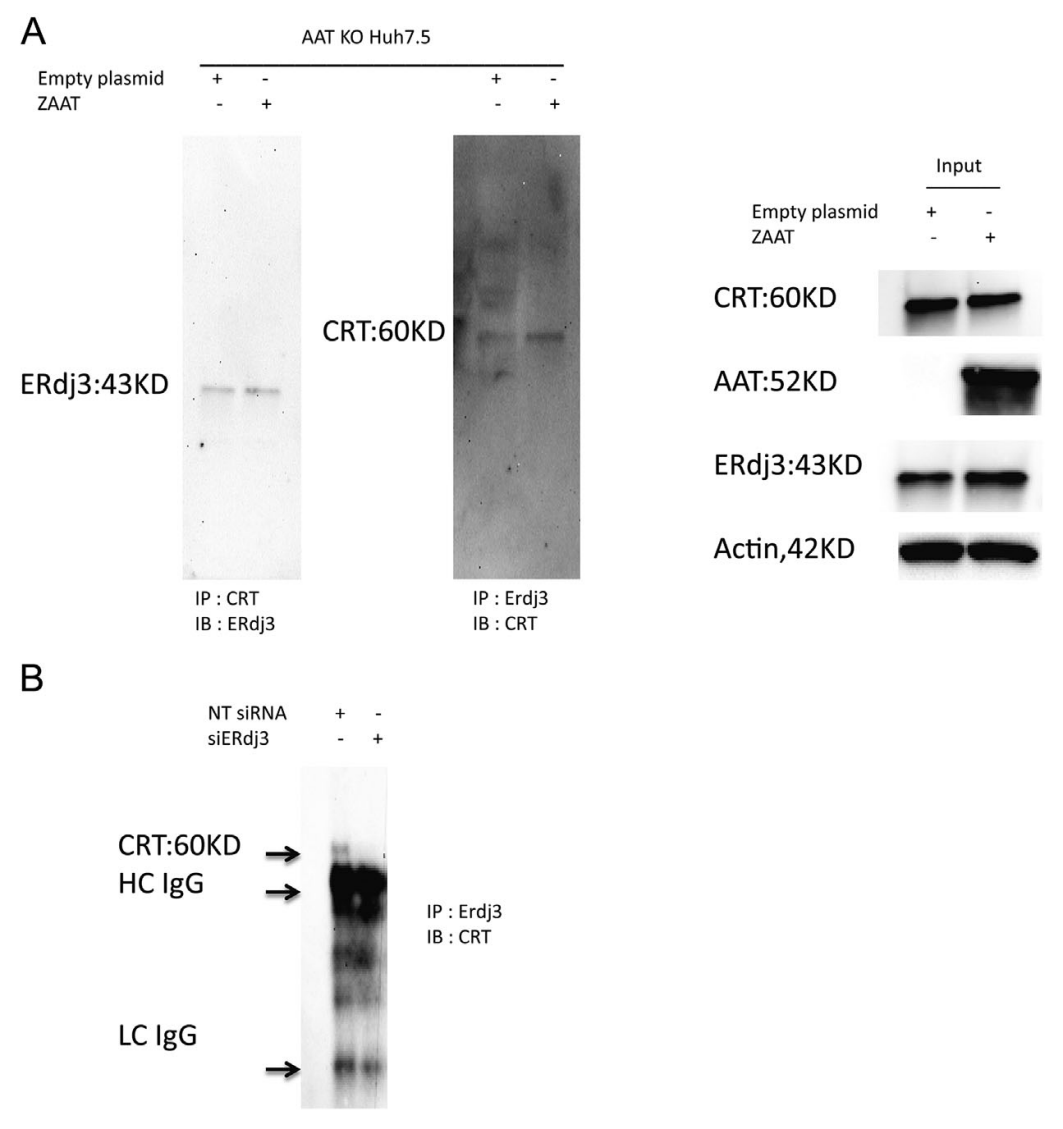
Interaction of ERdj3 and calreticulin in hepatocytes. (A) Empty vector or ZAAT plasmid was introduced to AAT KO Huh 7.5 cells. The ERdj3 association with calreticulin and vise versa was detected using antibody‐conjugated protein A Dynabeads. (B) NT siRNA or 20 µM of siERdj3 was introduced 24 hr post ZAAT transfection to AAT KO Huh 7.5 cells. The ERdj3 association with calreticulin was detected in NT siRNA‐treated samples using co‐IP.

ERdj3 is known to serve as a co‐factor for BiP association with unfolded proteins [Shen and Hendershot, 2005]. Because we were not able to detect ZAAT association with BiP chaperone and reduction of the concentration of ERdj3 in the ER results in enhanced ZAAT association with calreticulin instead, we investigated if there is any detectable interaction between ERdj3 and calreticulin. Using AAT KO Huh 7.5 cells transfected with empty or ZAAT plasmid calreticulin bound to ERdj3 and ERdj3 bound to calreticulin were detected by western blot analysis (Fig. 6A). In a separate experiment, calreticulin bound to ERdj3 was detected in cells transfected with NT siRNA and was not detectable in presence of siERdj3 (Fig. 6B).

## DISCUSSION

**Figure 7:**
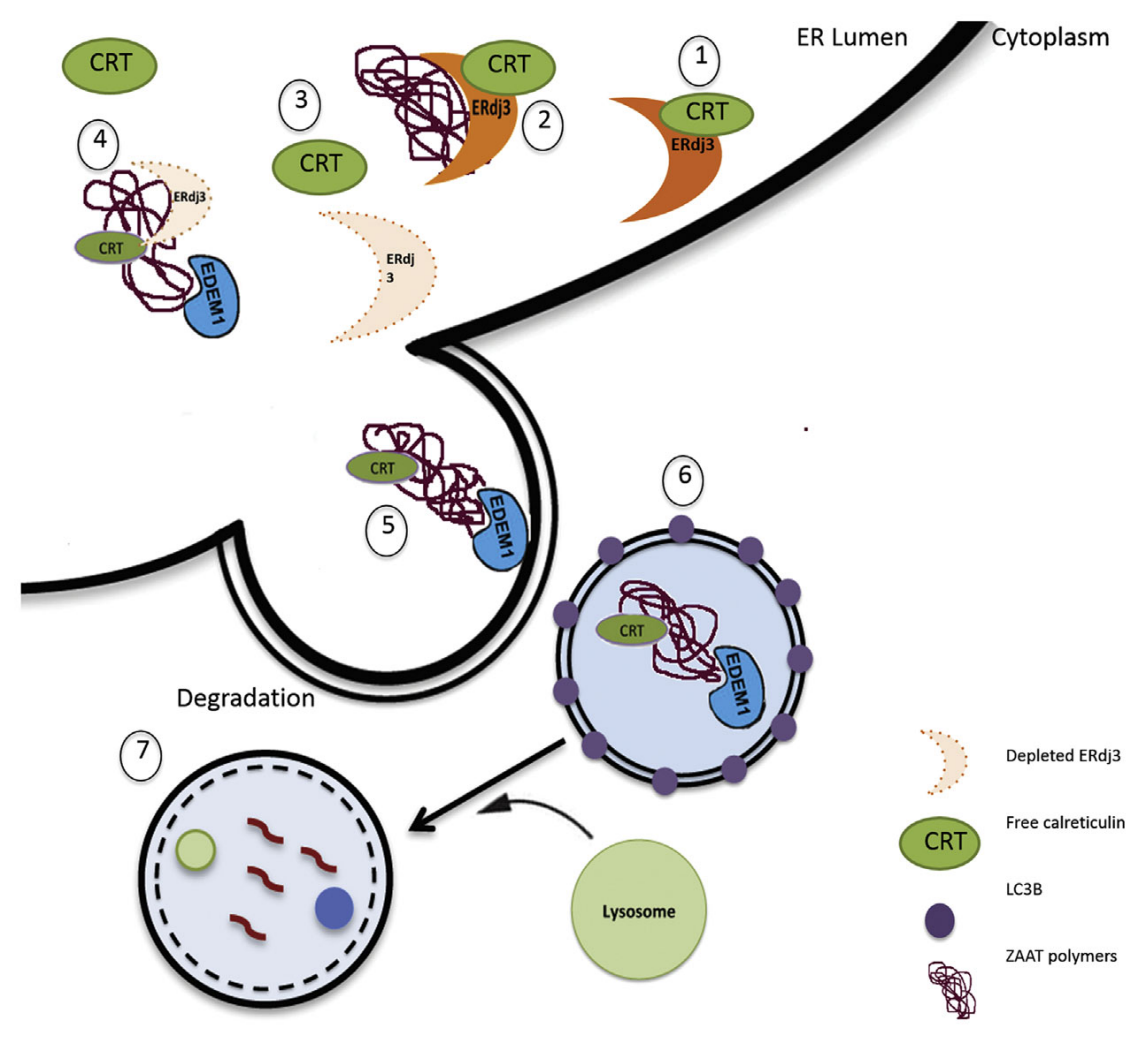
ERdj3 depletion causes autophagy pathway–mediated ZAAT degradation. (1) ERdj3 interacts and occupies calreticulin within the ER lumen. (2) ERdj3 binds to ZAAT polymers; there is no association between ZAAT and calreticulin. (3) Depletion of ERdj3 results in increasing free calreticulin in the ER lumen. (4) Calreticulin binds to ZAAT, followed by EDEM1 interaction with ZAAT. (5) Formation of autophagosomes containing ZAAT polymers bound to calreticulin and EDEM1. (6) LC3‐positive autophagosomes containing ZAAT polymers in cytosol. (7) Fusion of lysosomes with autophagosomes containing ZAAT polymers, followed by degradation of ZAAT polymers.

All proteins, regardless of the origin and individual characteristics are subject to the ER QC to ensure a correctly folded conformation. If the folding process fails, the misfolded protein is not transported to the final destination and is degraded [Ellgaard and Helenius, 2003]. In this study, our IP approach led us to discover new components of the ER QC system involved in AAT trafficking. We identified ERdj3 and calreticulin as two new AAT‐interacting proteins and trafficking network components. ERdj3 is a traditional HSP40 co‐chaperone that binds to unfolded proteins and transfers them to BiP [Otero et al., 2014; Genereux et al., 2015]. ERdj3 is co‐secreted with misfolded proteins to prevent their EC aggregation and proteotoxicity [Genereux and Wiseman, 2015]. Endogenous ERdj3 has been detected in conditioned media collected from a variety of human cell lines, including HepG2 and Huh7 cell lines [Genereux et al., 2015]. In addition, PiZZ individuals have plasma‐circulating ZAAT polymers that originate from their liver [Tan et al., 2014a]. The role of ERdj3 in regulating EC proteostasis led us to investigate whether ERdj3 co‐secretes with ZAAT and regulates ZAAT EC proteostasis. Because we detected secreted ERdj3 in the conditioned media of hepatocytes transfected with MAAT or ZAAT after 48 h of transfection (data not shown), we hypothesized that Erdj3 co‐secretes with ZAAT into the plasma. However, our ELISA experiment did not detect significant levels of ERdj3 in the plasma samples from normal individuals and those with AATD, and we were not able to support this hypothesis with ELISA (data not shown). Surprisingly, gene transfer–mediated overexpression of ERdj3 in ZAAT‐expressing hepatocytes resulted in increased ZAAT polymer formation. Previous data have indicated that overexpression of ERdj3 does not enhance the ER stress tolerance and that forced expression of ERdj3 might decrease the cell resistance to the ER stress [Nakanishi et al., 2004]. Both co‐IP and immunoflourscent experiments show that both ZAAT and MAAT associate and co‐localize with ERdj3. This observation strengthens ERdj3 as a novel protein that interacts with AAT and plays a regulatory role in AAT trafficking. Considering the result generated in this study and previous data, we investigated the role of ERdj3 in ZAAT trafficking. To better clarify this finding, we investigated the effect of silencing ERdj3 on the amount of IC ZAAT, using siRNA against ERdj3. Reducing the concentration of ERdj3 in the ZAAT‐expressing hepatocytes resulted in significant reduction of the total accumulated ZAAT in the cells and had no effect on the rate of ZAAT secretion into the media. In our metabolic labeling pulse‐chase analysis, an 80% reduction of ERdj3 expression in Huh 7.5 cells sped up the ZAAT clearance rate within the 4‐h chase period. Indeed, the duration of ZAAT degradation appears to be dependent on the concentration of ERdj3 in Huh 7.5 cells. In the same experiment for the M variant of AAT, ERdj3 depletion causes MAAT degradation because there is a significant reduction of IC MAAT, as well as EC MAAT. These results may argue that there is a degradation inhibitory role for ERdj3 in the AAT trafficking pathway that is beneficial for the normal variant and similarly results in ZAAT polymer formation in AATD. These results were confirmed with immunofluorescent experiments; compared with control cells, ZAAT‐expressing hepatocytes treated with siRNA against ERdj3 had 30% fewer polymers. Taken together, these data suggest that ERdj3 negatively regulates ZAAT degradation in the hepatocytes, which could be in agreement with previous reports showing that prolonged association of ERdj3 with substrates might delay their degradation [Shen and Hendershot, 2005] (Fig. 7).

To better define the pattern of increased ZAAT degradation, ZAAT clearance was persuade under Bafilomycin A1 and MG132 treatment in a 4‐h chase period. Interestingly, autophagy (to a greater extent) and proteasomes (to less extent) are both involved in siERdj3‐mediated ZAAT degradation, and inhibiting each pathway results in suppression of siERdj3‐mediated ZAAT degradation. In our immunoflourscent experiment, more green fluorescent autophagosomes appeared in the ZAAT globule‐containing hepatocytes treated with siERdj3. Moreover, in our IB experiment on density gradient, isolated cellular proteins revealed that siERdj3 co‐localized ZAAT with EDEM1 and calreticulin in Lamp1‐ and LC3B‐positive compartments. These ZAAT‐containing autolysosomes are located in the light‐density fraction of the cells and outside the ER. Thus, ERdj3 depletion may enhance the disposal of ZAAT by autophagy. These results reinforce previous data showing the involvement of the autophagy pathway in ZAAT degradation [Perlmutter, 2006; Kroeger et al., 2009; Marciniak and Lomas, 2010; Hidvegi et al., 2011]. Previous studies have shown that EDEM1 is an ERAD component involved in retro‐translocation of ERAD substrates. The surplus of EDEM1 may become deglycosylated and degraded by autophagy to prevent cytotoxicity caused by EDEM1 overexpression in the case of ER overload [Le Fourn et al., 2009]. We observed that endogenous EDEM1 appears to exit from the ER to the autolysosomes in both glycosylated and deglycosylated forms due to the presence of siERdj3. Furthermore, we observed that ZAAT co‐immunoprecipitated with EDEM1 and was degraded in the autolysosomes located in the light‐density fraction. This suggests that ERdj3 depletion enhances the formation of autophagosomes containing ZAAT and EDEM1. However, it could be hypothesized that the misfolded ZAAT is degraded accompanied by the surplus of EDEM1, whereas the ER load is diminished due to degradation of misfolded ZAAT. In our co‐IP experiment, there was more interaction between ZAAT and calreticulin through ERdj3 depletion. Calreticulin is an ER luminal chaperone that is involved with the folding of newly synthesized glycoproteins [Corbett et al., 2000]. Calreticulin, together with calnexin and ERp57, forms the calreticulin/calnexin cycle that efficiently suppresses the aggregation of glycosylated proteins within the ER [Michalak et al., 2009]. In our model, misfolded ZAAT segregates between a calreticulin folding pathway and ERdj3‐mediated ERAD pathway. While ERdj3 competes with calnexin in the ER [Tan et al., 2014b], ERdj3 and calreticulin may compete for the misfolded ZAAT in our model. ERdj3 depletion leads misfolded ZAAT to the calreticulin‐assisted folding pathway and allows calreticulin to bind to ZAAT. Because we were able to detect association between ERdj3 and calreticulin in our model, a reduced ER concentration of ERdj3 may cause more calreticulin accessibility for ZAAT to bind. ZAAT association with calreticulin suppresses ZAAT aggregation, resulting in subsequent EDEM‐1 association with ZAAT, and the complex is eventually degraded by autophagy. However, further studies will be required to clarify the precise mechanisms underlying ERdj3 depletion‐mediated ZAAT degradation through the calreticulin‐assisted folding pathway.

In conclusion, the present data demonstrate that DnaJ homolog ERdj3, a soluble resident ER glycoprotein, interacts with molecular chaperon calreticulin in the ER of Huh7.5 cells. In addition, we also demonstrated the interaction between ERdj3 and ZAAT and the inhibitory role of ERdj3 on ZAAT degradation. Moreover, the functional data collected support the concept that ERdj3 depletion substantially recruits the autophagy degradation machinery in an efficient way for disposal of ER‐ retained ZAAT. Our study indicates that increasing the ZAAT autophagy pathway by depleting ERdj3 while simultaneously enhancing the pro‐folding calreticulin pathway markedly inhibits ZAAT polymer aggregation within the ER of Huh7.5 cells. Because we did not observe any indication of UPR or decreased cell viability with this treatment, silencing ERdj3 may be a promising therapeutic approach for liver disease caused by AATD‐related toxic gain of function in hepatocytes.

## Supporting information

Additional supporting information may be found in the online version of this article at the publisher's web‐site.

Supporting Legends S1.
