# Supplementary material for: Erdj3 Has an Essential Role for Z Variant Alpha‐1‐Antitrypsin Degradation
Source: J Cell Biochem. 2017 Jun 20;118(10):3090–101. doi: 10.1002/jcb.26069 (PMC5575529; doi:10.1002/jcb.26069)
Supplement: Supplementary file 1 — Supporting Legends S1. [file JCB-118-3090-s001.docx]

Figure S1. **siERdj3 does not affect cell viability in the hepatocytes.** NT siRNA or 20 μM of siERdj3 was introduced 24 hours post ZAAT transfection to AAT KO Huh7.5 cells in 96-well plates, and live-dead assay was performed using live-dead viability/cytotoxicity kit. Data show no significant changes between the percentage of live and dead cells within three groups (n = 12) of treatment.

Figure S2. **ERdj3 interacts and co-localizes with MAAT in hepatocytes and prevents MAAT degradation.** (A) MAAT co-immunoprecipitated with ERdj3. AAT KO Huh7.5 cells were transfected with 0.1, 0.25, and 0.5 μg/mL of MAAT plasmid, followed by 3-hour incubation with 20 μM of Brefeldin A. The interaction between MAAT and ERdj3 was determined using co-IP. (B) ERdj3 co-localizes with RFP-tagged MAAT. RFP-MAAT transiently transfected cells were immunostained using anti-ERdj3 (Alexa 488; green), and nuclei were stained using DAPI (blue). (C) siERdj3 causes MAAT degradation in hepatocytes. NT siRNA or 20 μM of siERdj3 was introduced 24 hours post MAAT transfection to AAT KO Huh7.5 cells. IC and EC MAAT from NT siRNA– and siERdj3-treated samples were shown after pulse-chase radiolabeling.

Figure S3. **siERdj3 does not induce UPR in ZAAT-expressing hepatocytes.** (A) Silencing ERdj3 does not affect the protein level of major chaperones in the ER of hepatocytes. NT siRNA or 20 μM of siERdj3 was introduced 24 hours post ZAAT transfection to AAT KO Huh7.5 cells, and BiP, calnexin, calreticulin, and ERdj3 protein levels were detected by Western blot analysis. (B and C) Silencing ERdj3 does not affect RNA expression levels of UPR genes in the ER of hepatocytes. NT siRNA or siERdj3 (20 or 40 μM) were introduced 24 hours post ZAAT transfection to AAT KO Huh7.5 cells, and BiP, ATF4, spliced XBP1, CHOP, and AAT RNA levels were detected by qPCR analysis.
